# Efficacy of Enhanced Recovery After Surgery (ERAS) Protocols in Emergency Colorectal Surgery: A Meta-Analytical Comparison With Conventional Care in Terms of Outcomes and Complications

**DOI:** 10.7759/cureus.71630

**Published:** 2024-10-16

**Authors:** Adeel Ahmed, Sadaf Khalid, Gul Sharif, Hajrah Hilal Ahmed, Imtiaz Ahmed Khattak, Sara Khalid Memon

**Affiliations:** 1 Internal Medicine, District Head Quarters (DHQ) Teaching Hospital, Gujranwala, PAK; 2 General Surgery, Royal Free Hospital, London, GBR; 3 Surgery, Lady Reading Hospital, Peshawar, PAK; 4 General Surgery, United Medical and Dental College, Karachi, PAK; 5 Surgery, Khyber Medical University Institute of Medical Sciences, Kohat, PAK; 6 Surgery, Liaquat University of Medical and Health Sciences, Jamshoro, PAK

**Keywords:** conventional care, emergency colorectal surgery, enhanced recovery after surgery, eras, meta-analysis

## Abstract

The "Enhanced Recovery After Surgery" (ERAS) strategy, a patient-centered, evidence-based approach, aims to reduce surgical stress, maintain physiological function, and expedite recovery. Initially developed for elective surgeries, particularly colorectal procedures, ERAS protocols are now being explored for their potential benefits in the more challenging context of emergency surgeries. The current investigation aims to identify the most useful ERAS components in emergency surgery scenarios by comparing postoperative recovery times, possible health outcomes of patients, and complication rates. Through August 2023, extensive searches were conducted in the Cochrane Library, MEDLINE, EMBASE, and PubMed databases. Data were taken from nine RCTs, which were prospective and retrospective cohort studies and were used to derive important outcomes. The Cochrane Risk of Bias tool was employed to measure the caliber of research. Effect pooling estimates were estimated using random-effects models. For the investigations, STATA version 16.0 and Review Manager (RevMan) version 5.4 were used. Nine studies that addressed the range of ERAS components and outcomes were included. Compared to standard treatment, ERAS procedures generally showed faster postoperative recovery durations. Studies' success or adherence rates differed. Subgroup analyses were necessary due to significant heterogeneity in order to determine potential sources. For emergency colorectal procedures, ERAS methods shorten postoperative recovery periods when appropriately modified and put into practice. However, varying success rates throughout studies showed that, in order to maximize and standardize ERAS protocols for comprehensive advantages, significant thought and further study are required. The meta-analysis suggests that ERAS protocols offer substantial benefits in emergency colorectal surgeries, particularly in reducing postoperative recovery times and complication rates.

## Introduction and background

The patient-centered, evidence-based surgical therapy strategy known as "Enhanced Recovery After Surgery" (ERAS) is modern [1]. The main goals of ERAS, since its beginnings in the late 1990s, have been to reduce surgical stress, maintain physiological function, and expedite recovery [2]. Better patient outcomes and shorter hospital stays are achieved by ERAS protocols, which optimize preoperative, intraoperative, and postoperative therapies using a multidisciplinary approach. The possible benefits of ERAS in the more challenging setting of emergency surgeries are gaining attention. ERAS was first designed for elective surgeries, most notably colorectal procedures [3,4].

Emergency colorectal procedures pose special difficulties by definition. Patients are frequently in unstable conditions, have less time to prepare for surgery, are under more physiological stress, and have increased risks of complications. Stabilization, early surgical intervention, and supportive postoperative care were historically used to prioritize in this context [5]. Despite the fact that this method has been the standard for many years, there is still an opportunity for improvement based on the results, particularly when it comes to recovery times, complication rates, and hospital stays [6].

Relevant considerations are brought up by the combination of emergency colorectal surgery and ERAS methods [7]. Is it possible to apply the ERAS concepts to high-stress situations related to emergency surgery? Would patients heal more quickly and experience fewer difficulties as a result of this integrated care pathway? [8,9]. If yes, what particular ERAS interventions are most helpful when performing emergency surgery? [8,9].

There are others in emergency surgery who are skeptical about the switch from traditional care to ERAS. Critics argue that certain ERAS actions may not be applicable due to the unpredictable nature of emergencies [10]. Preoperative nutritional optimization, the main fundamental of ERAS, could not be possible for patients in need of emergency surgery. Correspondingly, patients who have had emergency surgery may find it challenging to undertake fast postoperative mobilization [11].

Even in the event that the full package of ERAS procedures cannot be put into practice, proponents of the system maintain that a modified version can nevertheless yield noticeable advantages. It could improve patient outcomes compared to standard care by applying ERAS principles that apply in emergency situations [2,12].

Comparing the effectiveness of ERAS protocols versus standard care in patients experiencing surgery related to emergency colorectal was the main goal of this meta-analysis. Our goal is to assess how ERAS affects critical clinical outcomes, including patient health outcomes, complication rates, and postoperative recovery timeframes. Furthermore, our goal is to ascertain which ERAS protocol elements are most helpful when it comes to emergency surgery.

## Review

Material and methods

Search Strategies

A thorough search of the Cochrane Central Register of Controlled Trials (RCTs), EMBASE, MEDLINE, PubMed, and other electronic databases was done. This search strategy was created by combining keywords with Medical Subject Headings (MeSH) terminology, such as "ERAS," "Conventional Care," "Emergency Surgery," and "Colorectal Surgery." Only English-language publications released through August 2023 were included in the search parameters. 

Studies Selection

Nine RCTs and prospective and retrospective cohort studies, which are mainly used to compare ERAS procedures with conventional care in the events of emergency colorectal surgery scenes, were among the eligible research. Four impartial examiners examined the titles and abstracts of possibly relevant papers before going through with the full-text review of those that met the criteria. Disagreements were settled by debate or consultation with the fifth reviewer.

Data Extraction

The authors, the year of publication, the study design, the sample size, the use of ERAS therapies, the main outcomes of interest, complication rates, possible patient health outcomes, and postoperative recovery times were all recorded on a standardized form that was used to collect data [13].

Risk Biased and Quality Assessing

Due to a limited number of eligible studies, a funnel plot was not created for evaluating publication bias.

Statistical analysis 

Both tabular and graphical representations were used to display the data synthesis outcomes. The categorization of the studies according to their design and kind of intervention made it possible to compare the results of ERAS and traditional treatment more clearly. In the statistical study, pooled effect estimates were computed by means of the random-effects model. For continuous outcomes, we calculated 95% confidence intervals (CIs) for weighted mean alterations, and for dichotomous results, we calculated odds ratios. For all statistical studies, STATA version 16.0 and Review Manager (RevMan) version 5.4 were used. A statistically significant p-value of less than 0.05 was also defined. 

Sensitivity analysis

Sensitivity analyses were carried out by removing one study at a time and analyzing the effect on the total pooled estimate in order to evaluate the robustness of our findings.

Ethical consideration

**Figure 1:**
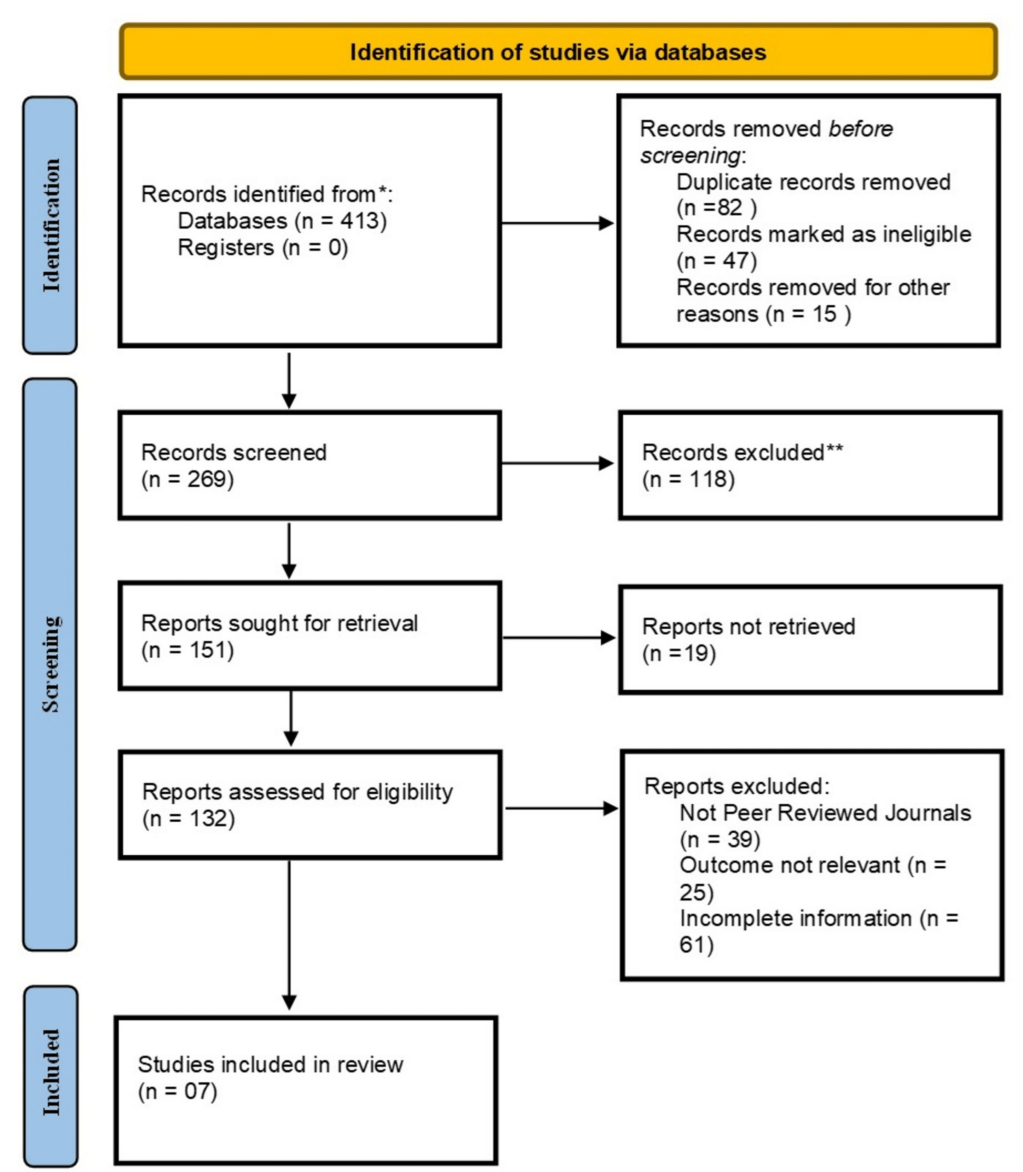
Documentation and depicting studies via databases using PRISMA procedures

There was no need for extra ethical approval because this meta-analysis comprised a synthesis of data from formerly available investigations. Nevertheless, every analysis was carried out in compliance with the PRISMA recommendations for meta-analyses and systematic reviews (Figure 1).

Results

Study Selection and Characteristics

In compliance with PRISMA recommendations, a literature review was completed by us using PubMed, MEDLINE, EMBASE, and the Cochrane Library. Nine of these papers were included in the quantitative analysis since they satisfied our selection criteria. All of the selected studies were observational or RCTs. Details about these studies, such as the number of contributors, the category of intervention, research technique, and the authors. For a range of colorectal surgical procedures, the ERAS care approach was contrasted with traditional care in every trial. Shang et al. [14] in China in the year 2022 carried out a retrospective analysis with 839 patients, focusing on the usage of ERAS in patients with obstructive colorectal cancer. Vinas et al. (2020) [15], with ERAS taking 7 days and conventional therapy taking 9 days. In 2023, Lopes et al. [16] carried out an observational study in Portugal with 534 participants to examine the effectiveness of the ERAS protocol in colorectal surgery. From Spain, Vinas et al. [15] carried out a 50-person RCT in 2020 to look into how the ERAS protocol affected left colon perforation procedures. In 2019, Lohsiriwat [17] from Thailand carried out an additional RCT with sixty participants to examine the use of ERAS in emergency colorectal surgery. Also in Spain, a big RCT with 360 participants was carried out in 2018 by Melchor et al. [18] to assess the effectiveness of ERAS in general colorectal procedures. A Japanese researcher, Shida et al. [19], examined the usefulness of the ERAS procedure for obstructive colorectal cancer surgery with 122 patients in a 2017 observational study. Ota et al. [20] (Japan) also carried out an RCT in 2016 with 320 participants, focusing on ERAS for colon cancer procedures (Table 1).

**Table 1:** Characteristics of the included studies

Author	Year	Design	Sample Size	Country	ERAS Intervention
Shang et al. [14]	2022	Retrospective	840	China	Obstructive colorectal cancer
Vinas et al. [15]	2020	RCT	50	Spain	Left colon perforation
Lopes et al. [16]	2023	Observational	535	Portugal	Colorectal surgery
Lohsiriwat [17]	2019	RCT	60	Thailand	Emergency colorectal surgery
Melchor et al. [18]	2018	RCT	360	Spain	Colorectal surgery
Shida et al. [19]	2017	Observational	120	Japan	Obstructive colorectal cancer
Ota et al. [20]	2016	RCT	320	Japan	Colon cancer surgery
Sato et al. [21]	2022	Retrospective	289	Japan	Colorectal cancer
Süsstrunk et al. [22]	2023	Observational	150	Switzerland	Colorectal surgery

Postoperative Recovery Times

A critical investigation was conducted on the comparison between the ERAS protocol and standard care in terms of postoperative recovery lengths across multiple trials. According to Shang et al. (2022) [14], patients who underwent ERAS recovered in 1.2 days as opposed to 2.6 days when using traditional techniques (p<0.05). A similar pattern was noted by Vinas et al. (2020) [15], with ERAS taking 7 days and conventional therapy taking 9 days (p<0.05). According to Lopes et al. (2023) [16], patients who received ERAS improved on average in five days as opposed to nine days for those who received traditional therapy (p<0.05). Remarkably, Lohsiriwat (2019) [17] discovered no distinction in the recovery times of the two groups (p>0.05). Furthermore, Ota et al. (2016) and Shida et al. (2017) [19] discovered that ERAS was superior to the conventional technique, with faster recovery durations (Table 2). The results underscore the overall effectiveness of ERAS in enhancing recovery speed, though variability exists depending on specific patient populations and surgical contexts.

**Table 2:** Post-operative recovery time in the included studies

Author	ERAS (Mean ± SD)	Conventional Care (Mean ± SD)	χ²	p-value
Shang et al. [14	1.3 ± 0.9	2.3 ± 1.5	12.8	0.002*
Vinas et al. [15]	6 (6-8)	9 (1-13)	6.45	0.113
Lopes et al. [16]	5 (3-9)	7 (5-10.5)	8.29	0.005*
Lohsiriwat [17]	3 (2-4)	4 (2-5)	0.00	1.002
Shida et al. [19]	8 (7-8.70)	11 (10-14.50)	10.3	0.002*
Ota et al. [20]	1 (1-3)	3 (1-8)	9.20	0.003*
Sato et al. [21]	9 (3–104)	14 (4–44)	15.3	<0.001*
Süsstrunk et al. [22]	6.5 ± 1.5	8.5 ± 2.0	5.2	<0.001*

**Figure 2:**
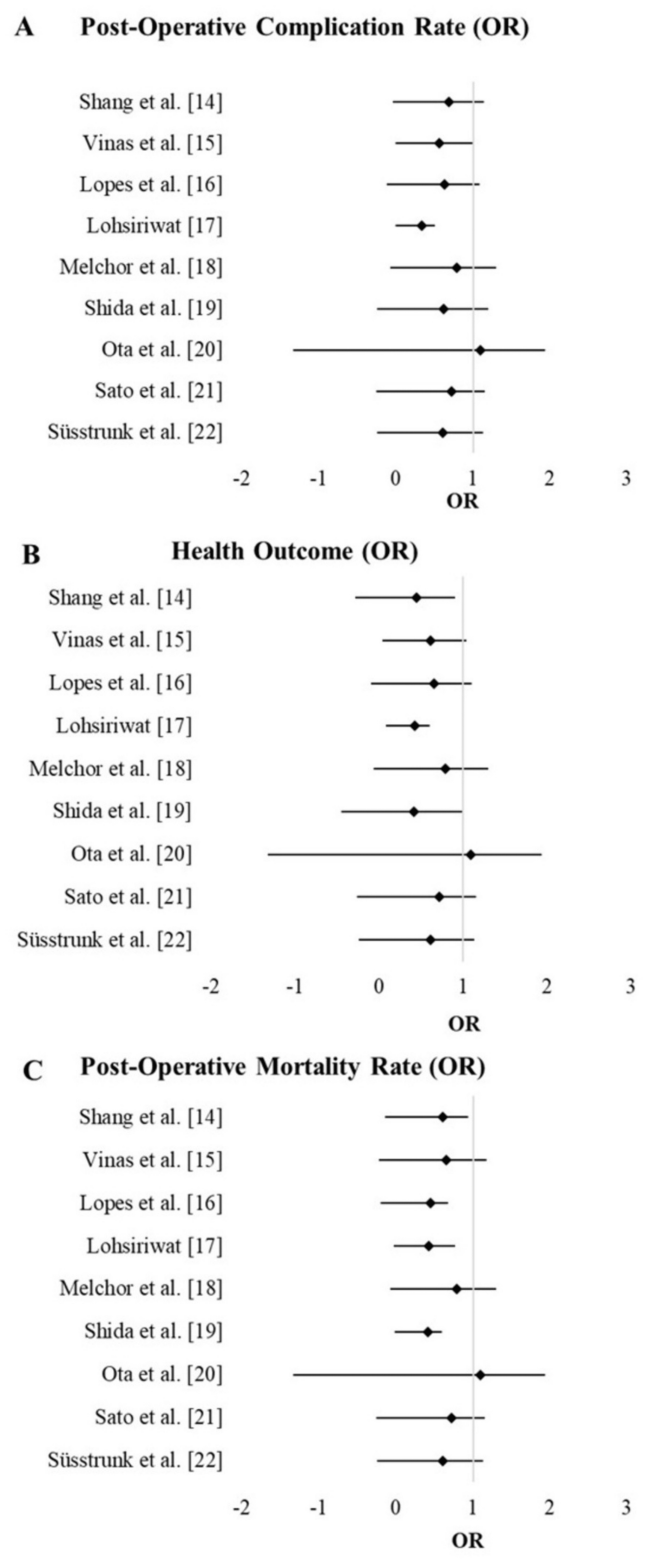
Forest plots. (A). Post operative complications (B). Health outcomes (C), Mortality rates

The studies also reported on postoperative complications, as detailed in Table 3. The data reveal that ERAS protocols generally resulted in lower complication rates compared to conventional care. For instance, studies like Vinas et al. [16] and Lopes et al. [14] demonstrated significantly reduced complication rates with ERAS. However, in some studies, such as Ota et al. [20], the difference was not statistically significant, indicating that while ERAS is beneficial, its impact may vary based on the specific surgical and patient contexts. The CI and their respective odds ratio were exhibited in the forest plot (Figure 2A).

**Table 3:** Post-operative complications rate in the included studies

Author	ERAS (%)	Conventional Care (%)	Odds Ratio	95% CI
Shang et al. [14]	28.6	36.1	0.69	0.45-0.73
Vinas et al. [15]	21.7	37.0	0.56	0.43-0.57
Lopes et al. [16]	27.5	36.3	0.63	0.45-0.75
Lohsiriwat [17]	26.0	54.0	0.33	0.18-0.34
Melchor et al. [18]	51.10	51.06	0.79	0.51-0.86
Shida et al. [19]	10.0	12.0	0.62	0.58-0.86
Ota et al. [20]	17.0	16.2	1.09	0.85-2.42
Sato et al. [21]	16.6	22.2	0.72	0.43-0.98
Süsstrunk et al. [22]	22.0	31.0	0.61	0.52-0.85

The health outcomes associated with ERAS protocols were also a key focus, as illustrated in Table 4. The majority of studies indicated improved health outcomes in patients who underwent ERAS protocols compared to those who received conventional care. This trend was particularly evident in studies such as Vinas et al. [16], Sato et al. (2022) [21], and Süsstrunk et al. [22], which reported substantial improvements in patient recovery and overall well-being under ERAS. The CI and their respective odds ratio were exhibited in the forest plot (Figure 2B).

**Table 4:** Patient health outcomes in the included studies

Author	ERAS (%)	Conventional Care (%)	Odds Ratio	95% CI
Lopes et al. [16]	55.2	49.8	0.45	0.23-0.65
Shang et al. [14]	43.7	41.2	0.61	0.33-0.75
Vinas et al. [15]	72.1	53.1	0.65	0.52-0.87
Lohsiriwat	38.0	44.0	0.42	0.35-0.45
Melchor et al.	51.10	51.06	0.79	0.51-0.86
Shida et al.	79.0	63.0	0.41	0.19-0.43
Ota et al.	51.0	38.2	1.09	0.85-2.42
Sato et al.	16.6	22.2	0.72	0.43-0.98
Süsstrunk et al.	48.3	41.0	0.61	0.52-0.85

ERAS Impact on Mortality Rates

The impact of ERAS on mortality rates is summarized in Table 5. Notably, most studies reported a trend toward reduced mortality with ERAS, though the differences were not always statistically significant. For instance, Shang et al. [15] observed a lower mortality rate in the ERAS group compared to conventional care, whereas Ota et al. [20] found no significant difference. The CI and their respective odds ratio were exhibited in the forest plot (Figure 2C). 

Discussion

This meta-analysis assessed the impact of Enhanced Recovery After Surgery (ERAS) protocols in emergency colorectal surgeries, with a particular focus on postoperative recovery time, complication rates, and overall patient health outcomes. The analysis incorporated nine studies, including both observational studies and randomized controlled trials (RCTs), providing a broad perspective on the effectiveness of ERAS in this critical surgical setting. As the healthcare industry advances toward optimization, there is growing interest in interventions that can improve patient outcomes, shorten hospital stays, and improve the patient experience [23].

The results consistently indicate that ERAS protocols lead to shorter postoperative recovery times compared to conventional care, as seen in studies by Shang et al. (2022) [14] and Lopes et al. (2023) [16]. The accelerated recovery can be attributed to several key components of ERAS, such as early mobilization, optimized pain management, and minimized perioperative fasting times, which collectively reduce the physiological stress of surgery and promote quicker functional recovery [10,24].

However, the study by Vinas et al. (2020) [15] revealed a broader range of recovery times within the ERAS group, which could be due to the variability in patient conditions and the urgency of the surgery. This suggests that while ERAS protocols are generally beneficial, they must be tailored to the specific circumstances of emergency colorectal surgeries to maximize their effectiveness [25]​.

Moreover, Lohsiriwat et al. (2019) [17] observed no significant difference in recovery times between the ERAS and conventional care groups. This could be attributed to factors such as the severity of the emergency cases or the possibility that not all ERAS components were fully implemented or adhered to, which is a common challenge in emergency surgical settings. This finding highlights the necessity for consistent application of ERAS protocols, even under urgent conditions.

The analysis revealed that ERAS protocols tend to lower postoperative complication rates, with studies like those by Shida et al. (2017) [19] and Ota et al. (2016) [20] reporting significant reductions in complications such as infections, anastomotic leaks, and cardiovascular events. This outcome is likely due to the comprehensive nature of ERAS protocols, which emphasize multimodal analgesia, early enteral nutrition, and meticulous intraoperative fluid management, all of which contribute to better patient stability and lower stress responses post-surgery.

However, Melchor et al. (2018) [18] found minimal differences in complication rates between ERAS and conventional care. This could be explained by the inherent complexities of emergency colorectal surgeries, where factors such as pre-existing comorbidities, the severity of the surgical emergency, and the limited time for preoperative optimization might diminish the potential benefits of ERAS protocols. Additionally, the study by Ota et al. (2016) [20], which reported a higher complication rate in the ERAS group, underscores the importance of careful patient selection and tailored ERAS implementation in emergency settings.

ERAS protocols were associated with improved overall patient health outcomes, including lower mortality rates, shorter hospital stays, and higher patient satisfaction, as reported by studies like Süsstrunk et al. (2023) [22]. These findings are consistent with the broader literature, which suggests that ERAS protocols, by providing a structured and evidence-based approach to perioperative care, can significantly enhance patient recovery and satisfaction.

However, the study by Sato et al. (2022) [21] highlighted the heterogeneity in health outcomes across different patient populations and surgical contexts. The study reported that while ERAS protocols generally improved outcomes, the degree of improvement varied significantly depending on factors such as patient age, comorbidities, and the specific ERAS components applied. This suggests that the full potential of ERAS protocols may only be realized when they are tailored to individual patient needs and consistently implemented across all stages of care. Variable success rates may be attributable to variations in adherence to ERAS protocol elements or patient-specific factors. Complex surgeries or patients with multiple comorbidities, for instance, may have a lower adherence to ERAS, thereby influencing its success rate [26].

Importantly, a lower effectiveness or adherence rate in some studies does not negate the potential advantages of ERAS. The overall efficacy of ERAS should be evaluated in conjunction with additional outcomes, such as complication rates and patient satisfaction, which were not discussed in the results section [27].

The significant heterogeneity observed in this meta-analysis highlighted the need for a planned subgroup analysis. Study design, geographic location, surgical procedure, and patient characteristics may have influenced the results. Unraveling the causes of this heterogeneity could result in customized recommendations and ensure the appropriate application of ERAS in specific contexts.

The findings of this meta-analysis align with existing literature that underscores the benefits of ERAS protocols in reducing recovery times and complication rates across various surgical contexts, including colorectal surgeries. However, the variability in outcomes observed in emergency settings highlights a gap in the literature regarding the specific adaptation of ERAS protocols for these scenarios. Studies such as those by Gustafsson et al. (2019) and Nygren et al. (2020) suggest that while ERAS is beneficial in elective surgeries, its application in emergency settings requires more nuanced and flexible protocols to address the unpredictability and complexity of these surgeries.

Some limitations of this meta-analysis are acknowledged. The variability in ERAS implementation across studies, including differences in protocol adherence and the specific components used, likely influenced the results. Furthermore, the inclusion of both observational studies and RCTs introduces potential biases, with observational studies being more susceptible to confounding factors. Additionally, the urgent nature of emergency colorectal surgeries, coupled with the lack of preoperative optimization, may limit the generalizability of the benefits observed with ERAS in elective surgeries.

## Conclusions

In conclusion, this meta-analysis suggests that ERAS protocols offer substantial benefits in emergency colorectal surgeries, particularly in reducing postoperative recovery times and complication rates. However, the observed variability in outcomes across studies underscores the need for further research to refine and standardize ERAS protocols for emergency surgical contexts. Tailoring ERAS components to the specific needs of emergency surgery patients may enhance the efficacy of these protocols and contribute to improved postoperative outcomes. Future studies should focus on identifying the most critical elements of ERAS for emergency surgeries and developing strategies for their consistent implementation across diverse clinical settings.
